# Plasma HULC as a Promising Novel Biomarker for the Detection of Hepatocellular Carcinoma

**DOI:** 10.1155/2013/136106

**Published:** 2013-05-22

**Authors:** Hui Xie, Hongwei Ma, Danqiu Zhou

**Affiliations:** ^1^Department of Laboratory Medicine, Zhengzhou People's Hospital, 33 Huanghe Road, Zhengzhou 450003, China; ^2^Henan Red Cross Blood Center, Institute of Transfusion Medicine, 9 Tongle Road, Zhengzhou 450012, China; ^3^Department of Laboratory Medicine, Jinshan Hospital, Shanghai Medical College, Fudan University, Shanghai 200540, China

## Abstract

Hepatocellular carcinoma (HCC) is a leading cause of cancer death in many Asian and African countries. Lack of early diagnosis tools is one of the clinical obstacles for effective treatment of HCC. Thus, enhanced understanding of the molecular changes associated with HCC is urgently needed to develop novel strategies for the diagnosis and treatment of this dismal disease. While aberrant expression of long noncoding RNAs (lncRNAs) has been functionally associated with certain cancers, the expression profiles and biological relevance of lncRNAs in HCC remain unclear. Highly upregulated in liver cancer (HULC) lncRNA has been implicated in the regulation of hepatoma cell proliferation. In this study, we demonstrate that HULC expression is significantly higher in HCC tumors compared to normal liver tissues. Among the tumor tissues, higher HULC expression is positively associated with Edmondson histological grades or with hepatitis B (HBV) positive status. Moreover, HULC lncRNA is detected with higher frequency in the plasma of HCC patients compared to healthy controls. Higher HULC detection rates are observed in the plasma of patients with higher Edmondson grades or with HBV+ status. These findings indicate for the first time that the expression of HULC in plasma can be used as a noninvasive promising novel biomarker for the diagnosis and/or prognosis of HCC.

## 1. Introduction

Hepatocellular carcinoma (HCC) is a leading cause of cancer death in many Asian and African countries [1, 2]. HCC causes estimated 662,000 deaths each year worldwide (“Cancer” World Health Organization), and about half of them occur in China. Although they can be small and slow growing, HCC tumors can be successfully treated by aggressive surgery, patients often lose the window for surgical resection due to the lack of effective tools for early diagnosis which results in very low 5-year survival rates. Therefore, to improve the prognosis of HCC, it is important and critical to develop specific and sensitive diagnostic biomarkers for HCC.

Noncoding RNAs (ncRNAs) consist of microRNAs, small interfering RNAs, and various classes of long noncoding RNAs (lncRNAs). NcRNAs play important regulatory roles in the development and progression of many diseases including cancers [3, 4].

LncRNAs, ranging from 200 to over 10,000 nucleotides, are abundantly transcribed by the mammalian genome [5, 6]. LncRNAs have been found to be dysregulated in a wide range of human diseases and disorders, including various types of cancer. For instance, lncRNAs PCGEM [7] and DD3 [8] are overexpressed in prostate cancer tissues, implicating that these lncRNAs may be involved in prostate tumorigenesis [9]. BC200 RNA overexpression has recently been correlated with the progression of breast tumors and proposed as a potential novel molecular marker for breast cancer [10]. Increased expression of the MALAT-1 gene indicates worse prognosis in lung cancer patients [11]. Together, these observations provide evidence and support for the potential roles of lncRNAs in tumor development and progression.

miRNAs in human plasma or serum have distinct expression signatures in different diseases including cancers. Therefore, serum miRNAs can serve as important diagnostic biomarkers for certain cancer types [12–15], such as colorectal, oral, and pancreatic cancers. However, lncRNA expressions in plasma or serum have not been investigated for their potential as potential novel biomarkers for HCC diagnosis or prognosis. 

Highly upregulated in liver cancer (HULC) was first identified from an HCC-specific gene library as a novel mRNA-like lncRNA which was markedly up-regulated in HCC [16] and is associated with the molecular pathogenesis of HCC. Aberrant expression of lncRNA HULC has been reported in HCC [17, 18], and its potential as a diagnostic biomarker has been proposed [15, 19]. However, the expression of HULC in the plasma of HCC patients has not been examined. In this study, we tested the hypothesis that lncRNA HULC is present in the plasma of HCC patients and can be used clinically as a biomarker to facilitate early diagnosis of HCC. We examined the expression level of HULC in HCC tumor tissues and liver tissues obtained from healthy volunteers and the matching plasma of these two groups using a real-time quantitative RT-PCR. Our results showed that HULC is markedly up-regulated in the HCC tumor tissues and in plasma of HCC patients.

## 2. Materials and Methods

### 2.1. Tissue and Blood Samples

Liver tissue samples from 20 healthy volunteers and tumor tissues from 30 HCC patients were collected from the Zhengzhou People's Hospital (Zhengzhou, China). Healthy volunteers had no history of HCC nor HBV infection and had normal physical examinations. Patients enrolled in the study were provided with written informed consent. Fresh tissue samples were frozen within 30 minutes after surgery and stored in liquid nitrogen until use. Tissue sections from each HCC sample were reviewed and classified by a pathologist. Chronic HBV infection is defined as HBV surface antigen positive for at least 6 months, HBV DNA detectable by PCR, and HBV infection-compatible results in a liver biopsy. 

Whole blood samples collected in EDTA tubes were centrifuged at 1,200 g for 10 min at 4°C to spin down the blood cells. The supernatants were transferred to microcentrifuge tubes and centrifuged at 12,000 g for 10 min at 4°C to completely remove cellular components. Plasma was then carefully collected, aliquoted, and stored at −80°C until use. Total RNA from 1 mL plasma was extracted using Trizol (Invitrogen) according to the manufacturer's instructions.

### 2.2. q-RT-PCR of HULC in HCC Tissues and Blood Samples

Reverse transcription reactions were carried out on 1 *μ*g total RNA using the PrimeScript RT reagent kit (TaKaRa BIO, Shiga, Japan). Random hexamer primers were used in the RT reactions. The real-time PCR was then performed using SYBR Premix DimerEraser kit (TaKaRa, Shiga, Japan). GAPDH was evaluated as a housekeeping gene for the qPCR reactions using the commercially available primers (TaKaRa, Shiga, Japan). The HULC qPCR primers used are forward, 5′-TCATGATGGAATTGGAGCCTT-3′; reverse, 5′-CTCTTCCTGGCTTGCAGATTG-3′. All the reactions were carried out on a Bio-Rad CFX-96 real-time PCR system (Bio-Rad, Hercules, CA) according to the manufactures' instructions. To ensure the accuracy of the amplifications, we included a negative control cell line HL-7702 which does not express HULC [20], and a positive control cell line Hep3B, which expresses high levels of HULC [20] in each run. To prepare the standards with 1%, 10%, 50%, and 100% of Hep3B cDNA load in a 50 ng/*μ*L background, serial dilutions of Hep3B cDNA with HL-7702 cDNA were made. The standard curve of HULC expression was plotted using the results of the serial dilution standards. The amplification efficiency of each standard reaction was confirmed to have an *r* ^2^ = 0.999. The concentrations of all cDNA samples in this study were within this standard range. Together, we confirmed that the HULC expression results are accurate. The expression level of HULC in each sample was normalized to that of the internal control GAPDH. The fold change of HULC expression in tumor samples versus healthy controls was calculated by the 2^−ΔΔCt^ method. 

### 2.3. Statistical Analysis

Statistical significances between groups were determined by two-tailed Student's *t*-test. The association between HULC expression and clinicopathological characteristics was analyzed using one-way ANOVA with bonferroni correction. All statistical analyses were done with STATA 10.0 (StataCorp LP, College Station, TX). A *P* value of <0.05 was considered significant. Receiver operating characteristics (ROC) curve was plotted to determine how well the expression level of HULC discriminated between tumor samples and healthy control samples.

## 3. Results

### 3.1. HULC lncRNA Expression Is Up-Regulated in HCC Tissues Compared to Normal Liver Tissues

**Figure 1:**
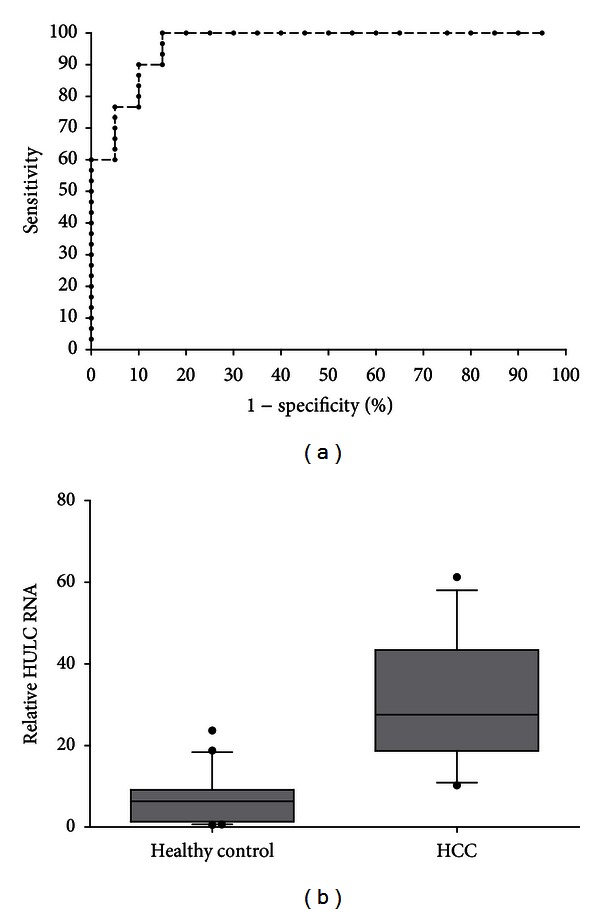
HULC is up-regulated in HCC tissues. Receiver-operating characteristic (ROC) curve (a) and box plots (b) of HULC RNA expression in HCC tissues and healthy control liver tissues. (a) The area under the ROC curve was 0.86 in distinguishing hepatocellular carcinoma versus healthy control. (b) HULC expression was examined in 20 healthy control liver tissues and 30 HCC tissues by qRT-PCR. Data were analyzed using the 2^−ΔΔCt^ method. The upper and lower limits of the boxes and the lines inside the boxes indicate the 75th and 25th percentiles and the median, respectively. The upper and lower horizontal bars denote the 90th and 10th percentiles, respectively.

To assess the potential clinical utility of HULC, its expression levels in both HCC tissues and healthy control liver specimens were analyzed. First, to determine the reaction efficiencies, standard curves were created using the qPCR results of serial dilutions of Hep3B cDNA which serves as a positive control for HULC expression. The linearity of real-time PCR was confirmed. Next, the receiver operating characteristics (ROC) analysis was used to evaluate the suitability of HULC expression to discriminate between the tumor and control samples. Total area under the curve (AUC) for HULC was 0.86 (Figure 1(a)), which suggests that HULC has adequate sensitivity and specificity to discriminate between tumor and control samples. Further, the expression of HULC in 30 HCC tumor samples and 20 healthy control liver tissue samples were determined. The results showed that the expression levels of HULC were markedly increased in HCC tissues compared to normal liver specimens (*P* < 0.01) (Figure 1(b)). 

### 3.2. HULC lncRNA Expression Is Associated with HCC Grades

**Figure 2:**
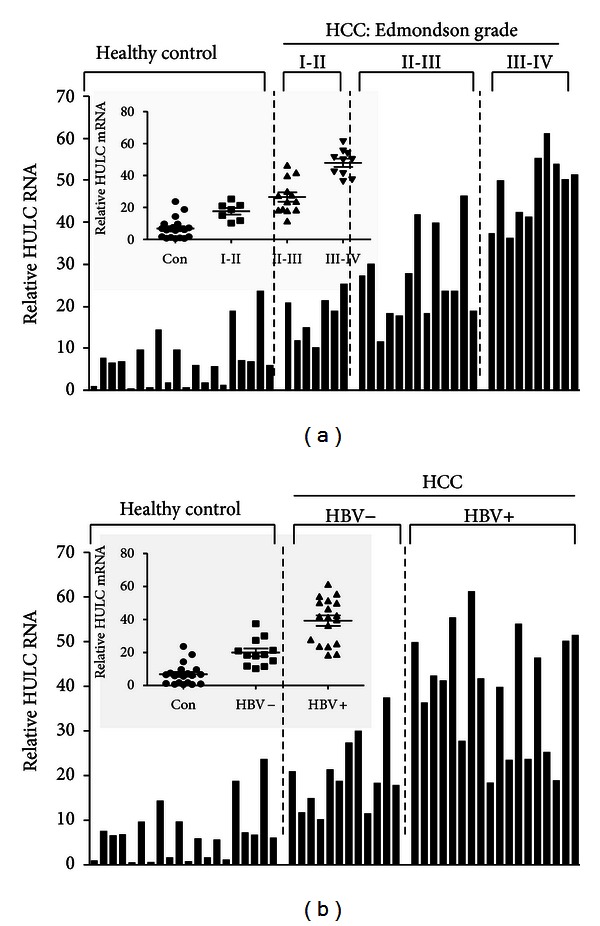
HULC lncRNA expression is associated with HCC Edmondson grades and HBV status. (a) Comparison of the relative levels of HULC in normal liver tissues and HCC tissues with different Edmondson grades. (b) Comparison of the relative levels of HULC in normal liver tissues and HCC tissues from HBV− and HBV+ patients.

To investigate whether the expression levels of HULC lncRNA in the HCC tumor tissues are associated with the disease severity, we analyzed the HULC expression in HCC tissues according to their Edmondson grades. The results revealed progressive up-regulation of HULC expression in HCC tissue samples from well-differentiated (Edmondson grades I-II) to undifferentiated lesions (Edmondson grades III-IV) (Figure 2(a)).

Chronic HBV infection is a major cause of HCC, and the multifunctional oncoprotein hepatitis B virus X (HBx) plays a crucial role in the development of HCC [21]. HBx-mediated up-regulation of HULC promotes the proliferation of hepatoma cells through the reduction of p18 [22]. The evidence prompted us to investigate whether there is any association between HULC expression and HBV infection status in HCC patients. We compared the expression of HULC lncRNA in HBV positive and negative HCC patients. HULC expression and HBV status were positively correlated in the HCC tissues (Figure 2(b)). HULC expression was higher in HCC tissue samples from HBV+ patients compared to those from HBV− patients (Figure 2(b)). The association of HULC expression with various clinicopathological parameters of the HCC patients was further analyzed statistically (Table 1). The median HULC expression levels were different in patients with different Edmondson histological grades or with different HBV status. The statistical analyses revealed a striking positive association between HULC expression levels and the HCC histological grades (*P* < 0.01) (Table 1). Statistical difference in HULC expression was also found between HBV positive and negative groups (*P* < 0.01) (Table 1).

### 3.3. HULC lncRNA Is Detected in the Plasma of HCC Patients

HULC RNA has been detected in peripheral blood cells of HCC patients [16]. Therefore, we investigated the potential of using HULC as a novel biomarker for HCC diagnosis. HULC expression levels were examined by qRT-PCR in plasma collected from healthy volunteers (*n* = 20) and HCC patients (*n* = 30) from whom the tissue samples were collected. HULC was detected in 63% (19/30) of the HCC patients, which was much higher than in the healthy control group (10%, 2/20) (Tables 2 and 3). Among the HCC patients, HULC detection frequencies increase with Edmondson grades (Table 2). The detection rates are 14%, 62%, and 100% for Edmondson grades I-II, II-III, and III-IV, respectively (Table 2). HULC was detected more frequently in the plasma of HBV+ HCC patients (90%) than in HBV− patients (25%) (Table 3). These observations indicate that the presence of HULC is an indication of HCC and also its progression. Therefore, the data support the clinical usage of HULC lncRNA as a potential biomarker for HCC diagnosis and prognosis.

## 4. Discussion

LncRNAs belong to a novel class of noncoding RNA molecules which are longer than 200 nucleotides [23]. A number of lncRNAs have been reported to control transcriptional alteration, implying that the difference of lncRNA profiling between normal and cancer cells may have regulatory roles in cancer progression instead of being the secondary effect of cancer transformation [24]. LncRNA expressions are strongly associated with cancer development and progression [24]. Thus, differential expression of lncRNAs in cancers may be used to facilitate cancer diagnosis, discover potential treatment targets, and improve prognosis. In this study, we demonstrate that lncRNA HULC is overexpressed in HCC tumor tissues compared to healthy liver tissues. Among the HCC patients, HULC expression is significantly higher in patients with higher Edmondson grades or with positive HBV status. These results indicate that HULC expression positively associates with the progression of HCC.

It has been widely reported that cancer-specific miRNAs are detectable in blood, sputum, urine, and other biological fluids of cancer patients. Therefore, miRNAs have been investigated as potential biomarkers for cancer diagnosis and prognosis. Likewise, lncRNAs have demonstrated utility as fluid-based markers of specific cancers. Serum and plasma harbor clinical discriminatory proteomic and transcriptomic biomarkers which can be easily assessed for clinical use. In the current study, HULC is detected in the plasma of HCC patients, and higher detection rates are found in the plasma of patients with higher Edmondson grades or positive HBV status. 

Our data suggest that HULC expression in HCC is likely to be associated with the aggressiveness and the progression of the tumor. While the prognostic power of HULC expression will obviously have to be substantiated by longitudinal analysis in prospective follow-up studies, our results represent a significant step towards establishing the utility of HULC expression as a prognostic indicator for HCC.

In conclusion, we have shown that HULC is differentially expressed in the tissues and plasma of the HCC patients compared with those of healthy controls. These findings indicate for the first time that the expression of HULC in plasma can be used as a novel and a rapid diagnostic and/or prognostic biomarker for HCC.

## Supplementary Material

Liver tissue samples were collected from 30 HCC patients, and 20 healthy controls. For each patient, we collected clinico-pathological information that included gender, age (year), tumor stage based on the Edmondson Grade system, and HBV status. Abbreviations: Gender: Male (M) or Female (F); HBsAg and HBV DNA: positive (+) or negative (−).

## Figures and Tables

**Table:** (a)

Normal			Tumor (Edmondson grade)								
*N* = 20			*N* = 30								
*n*	Median	*P*	I-II			II-III			III-IV		
			*n*	Median	*P*	*n*	Median	*P*	*n*	Median	*P*
20	6.81		7	20.64	<0.01	10	24.95	<0.01	13	47.97	<0.01

**Table:** (b)

Normal			Tumor (HBV)					
*N* = 20			*N* = 30					
*n*	Median	*P*	−			+		
			*n*	Median	*P*	*n*	Median	*P*
20	6.81		12	20.06	<0.01	18	39.32	<0.01

**Table 2:** HULC in the plasma of healthy controls and HCC patients.

lncRNA	Healthy control	HCC		
		I-II	II-III	III-IV
HULC	2/20 (10%)	1/7 (14%)	8/13 (62%)	10/10 (100%)

**Table 3:** HULC in the plasma of healthy controls and HCC patients with different HBV status.

lncRNA	Healthy control	HCC	
		HBV (−)	HBV (+)
HULC	2/20 (10%)	3/12 (25%)	16/18 (90%)
