# Supplementary material for: Plasma HULC as a Promising Novel Biomarker for the Detection of Hepatocellular Carcinoma
Source: Biomed Res Int. 2013 May 22;2013:136106. doi: 10.1155/2013/136106 (PMC3674644; doi:10.1155/2013/136106)
Supplement: Supplementary file 1 — Liver tissue samples were collected from 30 HCC patients, and 20 healthy controls. For each patient, we collected clinico-pathological information that included gender, age (year), tumor stage based on the Edmondson Grade system, and HBV status. Abbreviations: Gender: Male (M) or Female (F); HBsAg and HBV DNA: positive (+) or negative (−). [file 136106.f1.pdf]

Supplemental Table 1. The Characteristics of healthy individuals

Case No.	Gender	Age	HBsAg	HBV DNA	Collection
1	F	43	(-)	(-)	Biopsy
2	M	55	(-)	(-)	Biopsy
3	F	64	(-)	(-)	Biopsy
4	F	55	(-)	(-)	Biopsy
5	M	63	(-)	(-)	Biopsy
6	M	48	(-)	(-)	Biopsy
7	M	55	(-)	(-)	Biopsy
8	M	71	(-)	(-)	Biopsy
9	M	61	(-)	(-)	Biopsy
10	F	53	(-)	(-)	Biopsy
11	F	36	(-)	(-)	Biopsy
12	M	51	(-)	(-)	Biopsy
13	M	46	(-)	(-)	Biopsy
14	M	55	(-)	(-)	Biopsy
15	M	62	(-)	(-)	Biopsy
16	F	57	(-)	(-)	Biopsy
17	M	54	(-)	(-)	Biopsy
18	M	62	(-)	(-)	Biopsy
19	M	57	(-)	(-)	Biopsy
20	M	63	(-)	(-)	Biopsy

Supplemental Table 2. The Characteristics of HCC patients

Case No.	Gender	*Age*	*HBsAg*	HBV DNA	Edmondson Grade	*Collection*
21	M	38	(-)	(-)	I-II	Resection
22	M	59	(-)	(-)	I-II	Resection
23	F	54	(-)	(-)	I-II	Resection
24	M	42	(-)	(-)	I-II	Resection
25	M	46	(-)	(-)	I-II	Resection
26	M	61	(-)	(-)	I-II	Resection
27	M	59	(+)	(+)	I-II	Resection
28	M	57	(-)	(-)	II-III	Resection
29	M	51	(-)	(-)	II-III	Resection
30	M	56	(-)	(-)	II-III	Resection
31	M	39	(-)	(-)	II-III	Resection
32	M	53	(-)	(-)	II-III	Resection
33	M	49	(+)	(+)	II-III	Resection
34	M	52	(+)	(+)	II-III	Resection
35	M	62	(+)	(+)	II-III	Resection
36	F	61	(+)	(+)	II-III	Resection
37	M	62	(+)	(+)	II-III	Resection
38	M	61	(+)	(+)	II-III	Resection
39	M	47	(+)	(+)	II-III	Resection
40	M	60	(+)	(+)	II-III	Resection
41	F	64	(+)	(+)	III-IV	Resection
41	M	67	(+)	(+)	III-IV	Resection
42	M	74	(+)	(+)	III-IV	Resection
43	M	68	(+)	(+)	III-IV	Resection
44	M	70	(+)	(+)	III-IV	Resection
45	F	36	(+)	(+)	III-IV	Resection
46	M	42	(+)	(+)	III-IV	Resection
47	F	58	(+)	(+)	III-IV	Resection
48	M	64	(+)	(+)	III-IV	Resection
49	M	54	(+)	(+)	III-IV	Resection
50	F	62	(-)	(-)	III-IV	Resection
